# Beyond protein intake: does dietary fat intake in the year preceding pregnancy and during pregnancy have an impact on gestational diabetes mellitus?

**DOI:** 10.1007/s00394-021-02525-z

**Published:** 2021-03-04

**Authors:** Tian Qiao, Yue Chen, Ruonan Duan, Mengxue Chen, Hongmei Xue, Guo Tian, Yi Liang, Jieyi Zhang, Fang He, Dagang Yang, Yunhui Gong, Rong Zhou, Guo Cheng

**Affiliations:** 1 West China School of Public Health and West China Fourth Hospital, Sichuan University, Chengdu, People’s Republic of China; 2 West China School of Public Health and Healthy Food Evaluation Research Center, Sichuan University, Chengdu, People’s Republic of China; 3 College of Public Health, Hebei University, Baoding, People’s Republic of China; 4 Department of Clinical Nutrition, Affiliated Hospital of Guizhou Medical University, Guizhou Medical University, Guiyang, People’s Republic of China; 5 Sichuan Provincial Center for Disease Control and Prevention, No. 6 Middle School Road, Chengdu, People’s Republic of China; 6 West China Second University Hospital and Key Laboratory of Birth Defects and Related Diseases of Women and Children (Sichuan University) of Ministry of Education, Sichuan University, Chengdu, People’s Republic of China; 7 Laboratory of Molecular Translational Medicine, Center for Translational Medicine, Key Laboratory of Birth Defects and Related Diseases of Women and Children (Sichuan University), Ministry of Education, Department of Pediatrics, West China Second University Hospital, Sichuan University, Chengdu, Sichuan People’s Republic of China

**Keywords:** Gestational diabetes mellitus, Protein intake, Fat intake, Pregnant women, Cohort study

## Abstract

**Purpose:**

Studies regarding the association between dietary fat intake and gestational diabetes mellitus (GDM) are limited and provide conflicting findings. Thus, the study aims to examine the association of dietary fat intake in the year preceding pregnancy and during pregnancy with the risk of GDM, taking the relevance of dietary protein intake on GDM into consideration.

**Methods:**

A prospective study was conducted in 6299 singleton pregnancies, using the data from the Nutrition in Pregnancy and Growth in Southwest China (NPGSC). A validated food frequency questionnaire was used to assess dietary fat intake in the year preceding pregnancy and during the first and second trimesters of pregnancy. Logistic regression analysis was used to assess the prospective associations of dietary fat intake and the type and source of dietary fats in different time windows with GDM risk.

**Results:**

Higher intake of total fat [OR (95% CI): 2.21 (1.19–4.20), *P* = 0.02] during 12–22 weeks of gestation was associated with higher GDM risk. However, adjustment for animal protein intake greatly attenuated this association [OR (95% CI): 1.81 (0.93, 3.64), *P* = 0.11]. Total fat intake neither in the year preceding pregnancy nor during the early pregnancy was associated with GDM risk. Moreover, insignificant associations were observed between intakes of vegetable fat, animal fat, cholesterol, saturated fatty acid, monounsaturated fatty acid and polyunsaturated fatty acid one year before pregnancy and during the first and second trimesters and GDM risk.

**Conclusion:**

Our study indicated that dietary fat intake one year before pregnancy and across the two pregnancy trimesters preceding the diagnosis of GDM has no relevance on GDM risk among Chinese women, particularly those with normal BMI, low, or normal calorie intake.

## Introduction

Gestational diabetes mellitus (GDM) is one of the most common complications of pregnancy [1]. Worldwide, GDM is an increasing public health problem and its prevalence has continued to rise during the past two decades [2]. According to International Association of Diabetes and Pregnancy Study Groups (IADPSG) criteria, the prevalence of GDM in China is 18.3% in 2018, higher than that in USA and other developed nations [3]. Therefore, identification of modifiable risk factors for GDM may have direct effects on reducing the adverse health outcomes of both mother and offspring [4] and is of great public health significance among the Chinese population.

Dietary protein intake has been suggested to have an important influence on GDM risk [5, 6]. Our previous study indicated that higher dietary intakes of total protein and animal protein in mid-pregnancy were associated with an elevated GDM risk among Chinese women [7]. There are interactions between dietary protein and fat intake on dietary energy metabolism [8]. Furthermore, dietary fat may have a potential impact on glucose metabolism [9]. Although the pathogenesis of GDM remains largely unknown, the existing data suggested that the main defect of GDM is relatively diminished insulin secretion coupled with pregnancy-induced insulin resistance [10]. Notably, dietary fat may play a vital role in the development of insulin resistance, inhibiting insulin-stimulated glucose uptake by a physiological increase in plasma free fatty acid [11] or altering the enzyme activity [12] and gene expression [12, 13]. As the only macronutrient that directly affects postprandial blood glucose and long-term postprandial response, carbohydrate intake might be a significant dietary factor in the prevention of GDM. Several studies have found that a relatively low carbohydrate and high fat and protein intake may increase the risk of GDM [14, 15], after adjustment for confounders, including body mass index (BMI), which is an independent risk factor of GDM. It would thus be intriguing to explore whether dietary fat intake during prepregnancy and across the two pregnancy trimesters preceding the diagnosis of GDM have an impact on GDM development.

Evidence from observational studies [16–22] showed inconsistent findings on the relation of different types of fatty acids and sources of fat with GDM. However, the reviews focused on the associations between types of dietary fat and diabetes risk have indicated that the replacement of foods high in saturated fats with food sources of unsaturated (polyunsaturated and/or monounsaturated) fats could be favorable in the prevention of diabetes development [23] and plant sources of fat were also found to be a better choice than animal sources [24]. Hence, the importance of the type and source of dietary fats on the GDM development remains to be clarified.

Using prospective data from the Nutrition in Pregnancy and Growth in Southwest China (NPGSC), we thus aimed to investigate the association between dietary fat intake including specific fats as well as the source of fats (animal compared with vegetable fat) in the year preceding pregnancy and during early and mid-pregnancy and GDM risk. The relevance of dietary protein intake on GDM risk was taken special consideration.

## Method

### Study population

We used data from the NPGSC study, which is a prospective cohort study conducted among pregnant women and their children from 27 study centers (12 urban hospitals and 15 rural hospitals) of Sichuan, Yunnan, and Guizhou province in China, initiated in January 2014, aiming to investigate the relevance of maternal nutrition before and during pregnancy on health outcomes of mother and child, as described elsewhere [7].

The pregnant women were invited to the study center for interviews during their first visit for routine ultrasound examination at gestational weeks 9–11. To facilitate follow-up, participants who were cooperative and voluntary, who had lived in their current residence for at least 2 years, who signed an informed consent form were included in this cohort study. Generally, each visit included questionnaire, anthropometric measurements, and clinical examinations. The study was approved by the Ethics Committee of Sichuan University.

**Fig. 1:**
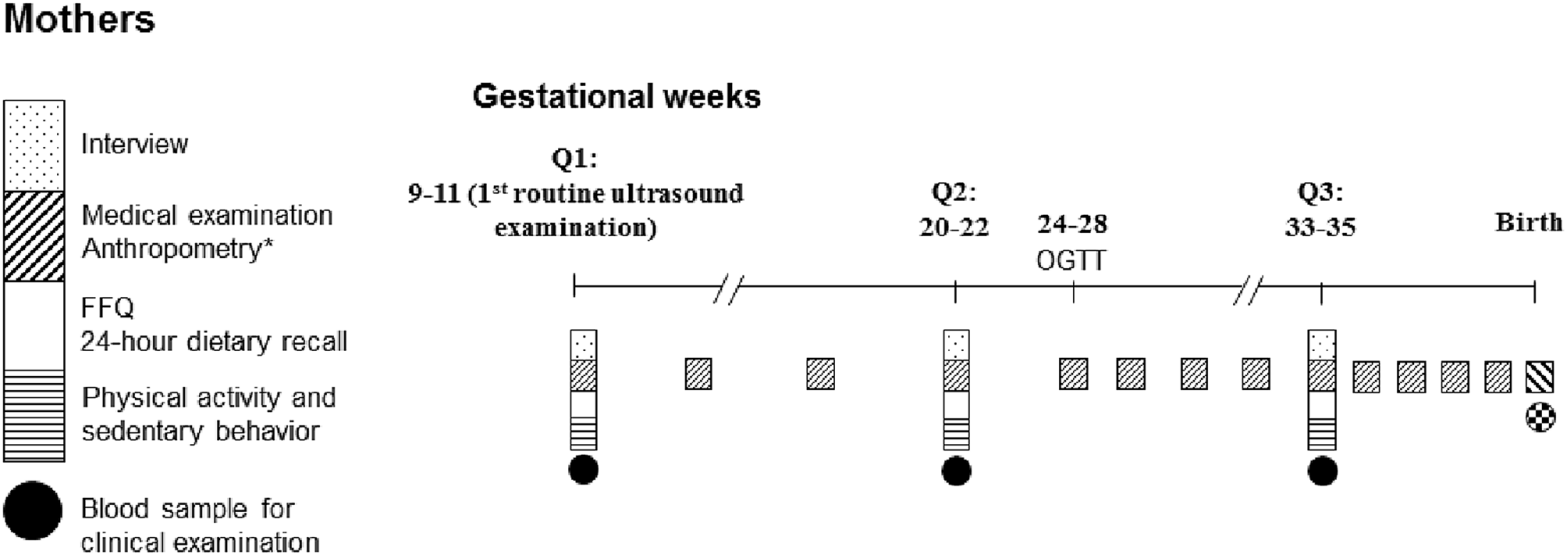
The study examination schedule

All participants were followed up for three times before women giving birth (the first routine ultrasound examination, Q1; gestational weeks 20–22, Q2; gestational weeks 33–35, Q3) and 8 times across offspring’s infancy and young childhood (Fig. 1). At Q1, participants were asked to complete a self-administered questionnaire approached by local nurses and trained interviewers, which collected information about their birth characteristics, demographic characteristics, educational and employment status, lifestyle characteristics (e.g., smoking behavior, alcohol consumption, tea/coffee consumption), medical history, annual family income, family history of chronic diseases, and so forth. In addition, each visit included face-to-face interviews by trained investigators about their diet [one food frequency questionnaire (FFQ) assessing usual dietary intake during the previous year before pregnancy and one 24-h dietary recall covering the consumption over the past 24 h] and physical activity (one questionnaire addressing physical activity and sedentary behavior over the past 12 months before pregnancy and from the start of the pregnancy, respectively). At Q2 and Q3, dietary data and physical activity were collected by trained investigators in face-to-face interviews with one FFQ assessing usual dietary intake over the past 12 weeks and one 24-h dietary recall each addressing intake over the past 24 h, as well as one physical activity questionnaire, respectively.

**Fig. 2:**
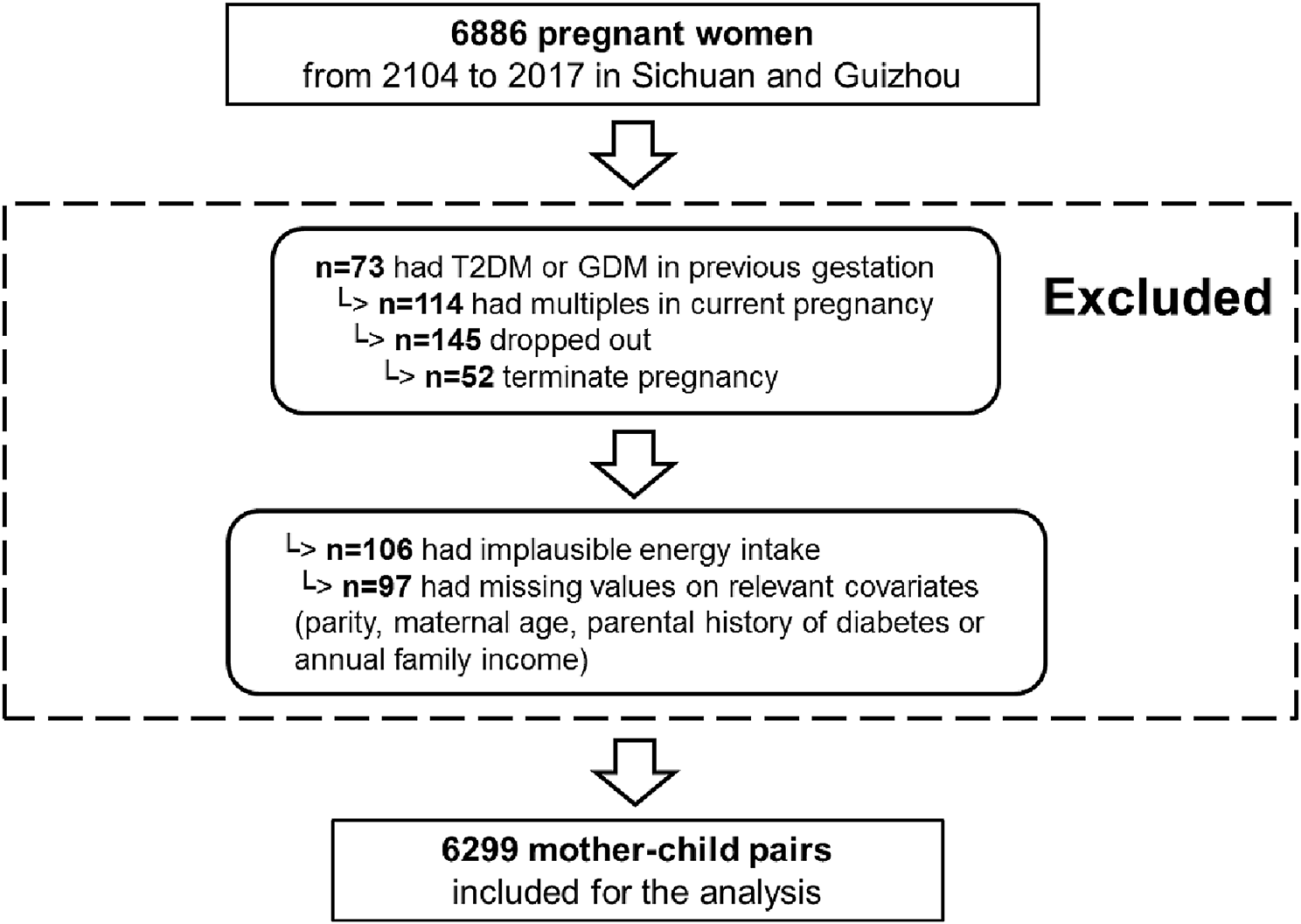
Study population sample from the NPGSC study cohort

The data used in this study were identified from the baseline survey of the prospective cohort study between 2014 and 2017. Participants in the present study lived in the Sichuan Provence and Guizhou Province (18 study centers: 8 urban hospitals and 10 rural hospitals). Initially, 6886 pregnant women were recruited. Of these, 587 were excluded: 73 diagnosed with the preexisting diabetes mellitus before pregnancy, 127 had stillbirth, multiple birth, or missing the first general questionnaire, 184 had incomplete information on three FFQs for the dietary intakes one year before pregnancy, at 1st trimester, or at 2nd trimester, 106 had implausible energy intake, and 97 had incomplete information on potential confounders. Therefore, the present analysis was based on a final sample of 6299 women (Fig. 2).

### Dietary intake assessment

At baseline, dietary intake 1 year before pregnancy was assessed using FFQ, that is, the present analysis was based on the dietary data collected by a 128-item FFQ. This FFQ was based on a validated questionnaire and additionally modified to the respective food groups [7]. At each assessment, participants were inquired to how often (daily, weekly, monthly, annually, never) on average they had consumed standard serving sizes of a specific type of food and or a common unit of weight in China (i.e., 1 liang = 50 g or natural units). Standardized tableware, including the bowls, plates, and glasses was provided for study respondents to improve the accuracy of the estimated serving sizes. Dietary intake data of each food and beverage reported by the FFQ were converted into grams per day and total energy and nutrients were calculated according to the continuously updated in-house nutrient database [25], which reflected the China Food Composition [26]. This nutrient database includes any food item ever recorded in previous studies of our institute, and is based on the information from standard nutrient tables, product labels, or recipe simulation of missing nutrient data for foods (e.g., new commercial food products) based on the labeled ingredients and national food tables.

### Clinical examinations

Self-reported pregravid weight was recorded on the day of registration. Trained investigators in each study center obtained body weight measurements according to standard procedures at enrollment and at regular intervals (in 4-week intervals from enrollment to week 25, every 2-week until week 33 weekly thereafter) to birth (Fig. 1). Body weight and maternal height (at enrollment) were measured with an ultrasonic instrument (Dingheng, Zhengzhou Province, China) to the nearest 0.1 kg and 0.1 cm, respectively. The information was recorded in the Medical Birth Registry.

The first ultrasound scan (Eub 5500, Hitachi; Eub 7500, Hitachi; Logiq E9, GE) measured in a standard manner by trained ultrasonographers on the day of registration and self-reported data on the last menstrual period were used for estimating the gestational age (GA), namely, if both measures were available and there was agreement (± 14 days) self-report data was used, otherwise, ultrasound data were used.

### Diagnosis of GDM

All participants underwent a 2-h 75-g oral glucose tolerance test (OGTT) in the morning after at least 8 h of fasting between 24 and 28 weeks of gestation. Venous plasma glucose at fasting, 1 and 2 h after the glucose load were measured by trained nurses. According to the IADPSG`s criteria, a diagnosis of GDM was made if one or more plasma glucose values of the following cutoff values were met: fasting plasma glucose ≥ 5.1 mmol/L or 1 h plasma ≥ 10.0 mmol/L or 2-h plasma glucose ≥ 8.5 mmol/L.

### Statistical analysis

SAS procedures (version 9.3, SAS Inc, Cary, NC) were used for all data analyses. All analyses were performed with a significance level at *P* < 0.05, except for interaction tests, in which *P* < 0.1 was considered significant in multivariable analysis. Preliminary analyses indicated no interactions between age and the relation of dietary fat intake with the GDM risk. Thus, the data from different ages were pooled for all analysis.

In this analysis, we conducted separate analyses using dietary data collected in the year preceding pregnancy and during the 1st trimester and 2nd trimester of pregnancy, aiming to differentiate the critical time window in which dietary fat intake impacts GDM onset. Dietary fat intakes in these three different periods were expressed each as residuals from their regression on energy intake. Multiple models of logistic regression were used to estimate the odds ratios (ORs) and 95% CIs of GDM risk in relation to energy-adjusted residuals of dietary fat intakes (into tertiles). Comparisons among tertiles of dietary fat intake were performed using an analysis of variance (ANOVA) for continuous variables and the chi-square test for categorical variables.

In the basic models, dietary fat intakes were the independent predictors. The following variables potentially affecting these associations were considered: maternal age, parity, residence (urban/rural), family history of diabetes, maternal education level (12 or more years of schooling; yes/no), maternal occupation (no, yes: part-time worker or full-time worker), monthly personal income (< 3000 CNY, 3000–6000 CNY, > 6000 CNY), pregravid BMI, gestational weight gain, physical activity, smoking/passive smoking before, or during pregnancy (never, past, current: 1–15, 16–24, or > 24 cigarettes/d), alcohol consumption before or during pregnancy (0, 0.1–9.9, 10.0–19.9, 20.0–29.9, ≥ 30 g/d), and intakes of carbohydrate and protein at the same time point of dietary fat. Each potential confounder was initially considered separately and included if it substantially modified the association of dietary fat with GDM or significantly predicted the outcome variable. Thus, maternal age, pregravid BMI and parity were retained in model 2. In the third step, we additionally adjusted for parental history of diabetes (yes or no), current smoking (combination of passive smoking and active smoking, yes or no), family income, total energy intake, gestational weight gain, and physical activity. In a further step, we controlled for confounding and/or mediation by carbohydrate intake (model IV a) and animal protein intake (model IV b), as they have been proposed to be relevant for GDM risk [6, 7, 14, 27]. Animal fat and vegetable fat were mutually adjusted for one another.

## Results

### General characteristics

General characteristics of the sample in this study stratified by dietary fat intake tertiles are shown in Table 1. In the present analysis, participants were on average 26.5 years old. No significant difference was found for age (*P* = 0.76), maternal educational level (*P* = 0.47), monthly personal income (*P* = 0.65) and family history of diabetes (*P* = 0.54) across all dietary fat intake groups. When compared with those consuming a diet with lower energy intake from dietary fat, participants in the highest energy intake from fat had a higher urbanization level (*P* < 0.001) and higher pregravid BMI (*P* = 0.04). In addition, higher energy intake from protein and lower energy intake from carbohydrate was observed in the highest dietary fat intake in the year preceding pregnancy and during the 1st trimester and 2nd trimester of pregnancy (*P* < 0.05).

**Table 1:** Characteristics by tertiles of fat intake (*n* = 6299)^1^

	Tertiles of fat intake			*p*
	T1	T2	T3	
Urban (*n* (%))	819 (39.0)	997 (47.5)	1214 (57.8)	< 0.001
Maternal age (years)	26.6 (3.8)	26.5 (3.8)	26.7 (3.6)	0.76
Pregravid BMI (kg/m^2^)	20.7 (2.6)	20.8 (2.1)	21.1 (2.5)	0.04
High educational level^2^ (*n* (%))	1110 (52.9)	1262 (60.1)	1294 (61.6)	0.47
High month income^3^ (*n* (%))	1104 (52.6)	1058 (50.4)	1119 (53.3)	0.65
Family history of diabetes (*n* (%))	455 (21.7)	509 (24.2)	528 (25.1)	0.54
Prepregnancy				
Smoking status (*n* (%))	85 (4.0)	51 (2.4)	34 (1.6)	0.38
Alcohol intake (*n* (%))	616 (29.3)	457 (21.8)	577 (27.5)	0.25
Physical activity^4^ (MET-h/wk)	20.4 (11.9)	17.7 (9.4)	17.4 (9.6)	0.19
Energy (kcal/d)	1571.7 (281.0)	1588.3 (286.9)	1838.7 (407.5)	< 0.001
Carbohydrate (% of energy)	60.8 (6.3)	52.5 (3.7)	42.5 (6.6)	< 0.001
Protein (% of energy)	15.1 (2.4)	15.8 (2.3)	15.7 (2.2)	0.04
Animal protein (% of energy)	7.4 (2.5)	9.4 (2.5)	11.2 (2.4)	< 0.001
Vegetable protein (% of energy)	7.4 (1.5)	6.2 (1.1)	5.1 (1.6)	< 0.001
Fat (% of energy)	25.0 (4.0)	31.6 (2.6)	40.7 (5.6)	< 0.001
Animal fat (% of energy)	19.2 (4.6)	26.4 (3.2)	36.2 (5.8)	< 0.001
Vegetable fat (% of energy)	5.7 (2.7)	5.2 (2.9)	4.5 (4.2)	< 0.001
Early pregnancy (0–11 weeks of gestation)				
Smoking status (*n* (%))	31 (1.5)	0 (0.0)	0 (0.0)	< 0.001
Alcohol intake (*n* (%))	40 (1.9)	40 (1.9)	40 (1.9)	0.99
Physical activity (MET-h/wk)	12.9 (7.2)	13.9 (9.3)	12.8 (6.0)	0.67
Gestational weight gain (kg)	1.0 (1.9)	1.2 (2.1)	1.1 (2.0)	0.21
Energy (kcal/d)	1526.1 (384.2)	1645.7 (410.6)	1761.9 (431.7)	< 0.001
Carbohydrate (%of energy)	60.9 (5.7)	51.5 (5.2)	44.6 (5.6)	< 0.001
Protein (% of energy)	14.7 (2.1)	16.4 (2.8)	15.9 (2.1)	< 0.001
Animal protein (% of energy)	7.2 (2.2)	10.0 (2.9)	10.9 (2.3)	< 0.001
Vegetable protein (% of energy)	7.2 (1.5)	6.3 (1.7)	5.2 (1.5)	< 0.001
Fat (% of energy)	24.7 (3.9)	32.4 (2.7)	38.8 (3.8)	< 0.001
Animal fat (% of energy)	18.1 (4.8)	25.8 (3.7)	32.5 (4.5)	< 0.001
Vegetable fat (% of energy)	6.7 (2.8)	6.6 (3.2)	6.3 (3.6)	0.53
Mid pregnancy (12–22 weeks of gestation)				
Smoking status (*n* (%))	0 (0.0)	0 (0.0)	0 (0.0)	1.00
Alcohol intake (*n* (%))	3 (0.1)	8 (0.4)	8 (0.4)	0.69
Physical activity (MET-h/wk)	16.2 (10.0)	16.5 (7.8)	18.6 (11.7)	0.19
Gestational weight gain (kg)	5.3 (2.2)	5.5 (2.6)	5.6 (2.4)	0.04
Energy (kcal/d)	1978.9 (447.0)	1989.1 (394.0)	1990.7 (499.6)	0.85
Carbohydrate (%of energy)	57.2 (6.7)	49.1 (4.4)	41.8 (5.1)	< 0.001
Protein (% of energy)	16.8 (2.8)	18.1 (2.7)	18.2 (2.2)	< 0.001
Animal protein (% of energy)	8.8 (3.0)	11.4 (2.6)	12.8 (2.1)	< 0.001
Vegetable protein (% of energy)	7.6 (1.6)	6.7 (1.4)	6.0 (1.6)	< 0.001
Fat (% of energy)	26.1 (3.6)	32.6 (2.8)	40.0 (3.7)	< 0.001
Animal fat (% of energy)	19.5 (4.1)	26.2 (3.0)	33.3 (4.9)	< 0.001
Vegetable fat (% of energy)	6.6 (2.3)	6.4 (2.6)	6.6 (3.4)	0.86

^1^Values are medians and intakes were calculated as the percentage of energy by tertiles

^2^School education at least 12 years

^3^Personal income per month at least ≥ 3000 RMB, which is moderate level among the general population in Southwest China

^4^Metabolic equivalent hours of activity per week

### Total and source of dietary fat intakes and the risk of GDM

The results of logistic regression analyses for the association between total and source of dietary fat intakes and GDM risk (Table 2) showed that intakes of animal fat and vegetable fat in the year preceding pregnancy and during pregnancy were not associated with risks for GDM (*P* > 0.05).

**Table 2:** Total and source of dietary fat intakes before and during pregnancy and risk of gestational diabetes (*n* = 6299 )^1^

	Tertiles of fat intake			*p*
	T1	T2	T3	
Prepregnancy				
Total fat				
Median (%energy/d)	26.9	32.4	40.2	
Model I	1.00	0.67 (0.36–1.25)	0.67 (0.36–1.25)	0.25
Model II	1.00	0.68 (0.36–1.28)	0.67 (0.35–1.25)	0.35
Model III	1.00	0.76 (0.39–1.46)	0.70 (0.36–1.33)	0.52
Model IVa	1.00	0.76 (0.39–1.45)	0.71 (0.37–1.35)	0.53
Model IVb	1.00	0.92 (0.46–1.82)	1.06 (0.50–2.31)	0.92
Animal fat				
Median (%energy/d)	18.7	26.2	34.9	
Model I	1.00	0.57 (0.30–1.07)	0.68 (0.36–1.25)	0.19
Model II	1.00	0.58 (0.31–1.10)	0.67 (0.36–1.24)	0.21
Model III	1.00	0.69 (0.35–1.36)	0.69 (0.36–1.29)	0.41
Model IVa	1.00	0.68 (0.34–1.35)	0.68 (0.36–1.28)	0.40
Model IVb	1.00	0.87 (0.42–1.79)	1.08 (0.50–2.37)	0.83
Vegetable fat				
Median (%energy/d)	3.9	6.3	9.8	
Model I	1.00	1.11 (0.59–2.11)	1.17 (0.62–2.21)	0.89
Model II	1.00	1.15 (0.61–2.20)	1.18 (0.62–2.23)	0.86
Model III	1.00	1.59 (0.79–3.22)	1.40 (0.71–2.79)	0.41
Model IVa		1.61 (0.80–3.27)	1.47 (0.74–2.95)	0.38
Model IVb	1.00	1.32 (0.64–2.72)	1.00 (0.48–2.11)	0.65
Early pregnancy (0–11 weeks of gestation)				
Total fat				
Median (%energy/d)	25.4	32.1	37.3	
Model I	1.00	1.16 (0.63–2.15)	1.00 (0.53–1.87)	0.86
Model II	1.00	1.16 (0.63–2.15)	0.98 (0.52–1.84)	0.84
Model III	1.00	1.17 (0.63–2.19)	0.88 (0.46–1.68)	0.69
Model IVa	1.00b	1.17 (0.62–2.22)	0.89 (0.46–1.69)	0.68
Model IVb	1.00	1.15 (0.59–2.24)	0.86 (0.42–1.79)	0.68
Animal fat				
Median (%energy/d)	19.2	27.0	33.5	
Model I	1.00	0.90 (0.48–1.70)	1.27 (0.69–2.34)	0.53
Model II	1.00	0.89 (0.47–1.69)	1.25 (0.68–2.31)	0.55
Model III	1.00	0.89 (0.46–1.71)	1.14 (0.61–2.14)	0.75
Model IVa	1.00	0.90 (0.46–1.74)	1.15 (0.61–2.17)	0.75
Model IVb	1.00	0.91 (0.46–1.82)	1.18 (0.57–2.45)	0.74
Vegetable fat				
Median (%energy/d)	4.0	6.2	9.8	
Model I	1.00	0.86 (0.46–1.60)	1.00 (0.54–1.84)	0.86
Model II	1.00	0.87 (0.46–1.61)	1.02 (0.55–1.89)	0.85
Model III	1.00	0.95 (0.50–1.80)	1.04 (0.56–1.94)	0.96
Model Iva	1.00	0.94 (0.49–1.81)	1.03 (0.54–1.96)	0.96
Model IVb	1.00	0.95 (0.50–1.81)	1.04 (0.56–1.96)	0.96
Mid pregnancy (12–22 weeks of gestation)				
Total fat				
Median (%energy/d)	27.2	32.6	39.4	
Model I	1.00	1.06 (0.56–2.01)	1.95 (1.08–3.58)	0.04
Model II	1.00	1.08 (0.57–2.08)	1.97 (1.09–3.62)	0.04
Model III	1.00	1.19 (0.61–2.33)	2.24 (1.21–4.24)	0.02
Model IVa	1.00	1.17 (0.60–2.30)	2.21 (1.19–4.20)	0.02
Model IVb	1.00	1.02 (0.51–2.06)	1.81 (0.93–3.64)	0.11
Animal fat				
Median (%energy/d)	20.1	25.9	32.7	
Model I	1.00	0.90 (0.48–1.69)	1.49 (0.83–2.69)	0.20
Model II	1.00	0.93 (0.49–1.76)	1.49 (0.83–2.70)	0.24
Model III	1.00	0.98 (0.51–1.90)	1.64 (0.90–3.01)	0.16
Model IVa	1.00	0.96 (0.50–1.87)	1.60 (0.86–3.01)	0.18
Model IVb	1.00	0.84 (0.42–1.65)	1.26 (0.64–2.48)	0.44
Vegetable fat				
Median (%energy/d)	3.7	6.0	9.1	
Model I	1.00	1.95 (1.06–3.65)	1.51 (0.81–2.87)	0.11
Model II	1.00	1.98 (1.08–3.73)	1.50 (0.80–2.87)	0.10
Model III	1.00	1.92 (1.02–3.69)	1.52 (0.79–2.94)	0.14
Model IVa	1.00	1.98 (1.05–3.84)	1.64 (0.84–3.26)	0.11
Model IVb	1.00	2.02 (1.06–3.92)	1.68 (0.87–3.31)	0.10

^1^Values are medians and intakes were calculated as the percentage of energy by tertiles. OR and 95% CI calculated by logistic regression. Model I was crude model. Model II was additionally adjusted for age, pregravid BMI, and parity. Model III was additionally adjusted for family history of diabetes (yes or no), current smoking (yes or no), family income, total energy intake, gestational weight gain, and physical activity. Model IV a was additionally adjusted for carbohydrate intake. Model IV b was additionally adjusted for animal protein intake

Higher intake of total fat during 12–22 weeks of gestation resulted in a significant higher GDM risk in initial model (*P* = 0.04). Even after adjustment for maternal age, pregravid BMI, parity, parental history of diabetes, current smoking, family income, total energy intake, gestational weight gain, physical activity, and carbohydrate intake (model IV a), women with highest total fat intake had an approximately 121% higher odds to those in the lowest tertile (95% *CI*: 1.19, 4.20, *P* = 0.02). However, this association was considerably attenuated once animal protein intakes (model IV b) were adjusted for (95% CI: 0.93, 3.64, *P* = 0.11), but did improve the fit of the final model. Moreover, total fat intake neither in the year preceding pregnancy nor during the early pregnancy was associated with GDM risk.

### Specific dietary fat and cholesterol intakes and the risk of GDM

The results of logistic regression analyses of specific dietary fat and cholesterol intakes and the risk of GDM are shown in Table 3. We did not observe the significant association between intakes of total dietary cholesterol (*P* = 0.13), saturated fatty acid (SFA) (*P* = 0.23), monounsaturated fatty acid (MUFA) (*P* = 0.16), and polyunsaturated fatty acid (PUFA) (*P* = 0.92) one year before pregnancy and during the first and second trimesters and GDM risk in fully adjusted models including both dietary and nondietary covariates.

**Table 3:** Specific dietary fat and cholesterol intakes before and during pregnancy and risk of gestational diabetes (*n* = 6299)^1^

	Tertiles of fat intake			*p*
	T1	T2	T3	
Prepregnancy				
Cholesterol				
Median (mg/d)	231.4	445.4	535.7	
Model I	1.00	0.78 (0.42–1.45)	0.66 (0.35–1.24)	0.43
Model II	1.00	0.76 (0.41–1.42)	0.64 (0.34–1.21)	0.38
Model III	1.00	0.74 (0.39–1.40)	0.68 (0.35–1.30)	0.46
Model IVa	1.00	0.74 (0.39–1.41)	0.67 (0.35–1.29)	0.45
Model IVb	1.00	0.95 (0.48–1.90)	1.04 (0.48–2.25)	0.97
Saturated fatty acid				
Median (g/d)	7.6	11.4	17.1	
Model I	1.00	0.75 (0.40–1.38)	0.60 (0.31–1.12)	0.27
Model II	1.00	0.74 (0.39–1.37)	0.58 (0.30–1.10)	0.24
Model III	1.00	0.91 (0.48–1.72)	0.62 (0.31–1.19)	0.33
Model IVa	1.00	0.90 (0.47–1.71)	0.62 (0.32–1.20)	0.35
Model IVb	1.00	1.15 (0.58–2.32)	0.91 (0.42–2.00)	0.80
Monounsaturated fatty acid				
Median (g/d)	9.5	14.6	22.1	
Model I	1.00	1.05 (0.57–1.93)	0.65 (0.33–1.23)	0.29
Model II	1.00	1.04 (0.56–1.91)	0.63 (0.33–1.22)	0.28
Model III	1.00	1.22 (0.65–2.30)	0.67 (0.34–1.32)	0.21
Model IVa	1.00	1.22 (0.65–2.30)	0.68 (0.34–1.35)	0.25
Model IVb	1.00	1.51 (0.78–2.98)	0.98 (0.45–2.13)	0.33
Polyunsaturated fatty acid				
Median (g/d)	5.0	8.6	13.9	
Model I	1.00	0.63 (0.33–1.18)	0.71 (0.38–1.31)	0.32
Model II	1.00	0.65 (0.34–1.21)	0.70 (0.37–1.29)	0.34
Model III	1.00	0.76 (0.37–1.56)	0.78 (0.39–1.56)	0.71
Model IVa	1.00	0.78 (0.37–1.61)	0.81 (0.40–1.64)	0.77
Model IVb	1.00	0.81 (0.39–1.67)	0.82 (0.41–1.64)	0.81
Early pregnancy (0–11 weeks of gestation)				
Cholesterol				
Median (mg/d)	214.5	430.4	539.8	
Model I	1.00	0.90 (0.48–1.69)	1.10 (0.60–2.03)	0.82
Model II	1.00	0.91 (0.48–1.70)	1.08 (0.59–2.00)	0.85
Model III	1.00	0.88 (0.46–1.67)	1.14 (0.61–2.13)	0.73
Model IVa	1.00	0.88 (0.46–1.67)	1.14 (0.61–2.13)	0.72
Model IVb	1.00	0.91 (0.46–1.80)	1.26 (0.56–2.89)	0.67
Saturated fatty acid				
Median (g/d)	7.3	11.2	17.0	
Model I	1.00	1.46 (0.80–2.69)	0.90 (0.47–1.71)	0.25
Model II	1.00	1.44 (0.79–2.67)	0.89 (0.46–1.70)	0.26
Model III	1.00	1.54 (0.83–2.91)	0.82 (0.42–1.59)	0.13
Model IVa	1.00	1.55 (0.82–2.93)	0.82 (0.42–1.61)	0.13
Model IVb	1.00	1.50 (0.78–2.95)	0.78 (0.37–1.67)	0.13
Monounsaturated fatty acid				
Median (g/d)	9.8	14.1	21.1	
Model I	1.00	0.95 (0.52–1.76)	0.91 (0.49–1.68)	0.95
Model II	1.00	0.94 (0.50–1.73)	0.90 (0.49–1.68)	0.94
Model III	1.00	0.93 (0.50–1.74)	0.84 (0.44–1.58)	0.86
Model IVa	1.00	0.94 (0.50–1.75)	0.84 (0.44–1.58)	0.85
Model IVb	1.00	0.92 (0.48–1.74)	0.82 (0.42–1.60)	0.84
Polyunsaturated fatty acid				
Median (g/d)	5.1	8.6	14.6	
Model I	1.00	0.95 (0.51–1.78)	1.05 (0.57–1.95)	0.95
Model II	1.00	0.96 (0.51–1.79)	1.06 (0.57–1.97)	0.95
Model III	1.00	1.04 (0.55–2.00)	1.08 (0.58–2.03)	0.97
Model IVa	1.00	1.04 (0.55–1.99)	1.08 (0.57–2.05)	0.97
Model IVb	1.00	1.04 (0.55–1.99)	1.08 (0.58–2.04)	0.97
Mid pregnancy (12–22 weeks of gestation)				
Cholesterol				
Median (mg/d)	255.4	449.3	558.7	
Model I	1.00	2.03 (1.08–3.90)	2.03 (1.08–3.90)	0.052
Model II	1.00	2.02 (1.08–3.89)	2.02 (1.08–3.89)	0.052
Model III	1.00	2.27 (1.18–4.50)	2.28 (1.19–4.52)	0.03
Model IVa	1.00	2.25 (1.17–4.47)	2.31 (1.20–4.59)	0.03
Model IVb	1.00	2.02 (1.03–4.09)	1.72 (0.79–3.89)	0.13
Saturated fatty acid				
Median (g/d)	7.7	11.5	18.0	
Model I	1.00	0.86 (0.45–1.61)	1.55 (0.87–2.79)	0.12
Model II	1.00	0.87 (0.46–1.64)	1.55 (0.87–2.80)	0.13
Model III	1.00	0.92 (0.47–1.81)	1.70 (0.92–3.17)	0.10
Model IVa	1.00	0.91 (0.46–1.79)	1.65 (0.88–3.13)	0.11
Model IVb	1.00	0.79 (0.39–1.57)	1.35 (0.71–2.62)	0.23
Monounsaturated fatty acid				
Median (g/d)	10.1	14.6	22.2	
Model I	1.00	1.17 (0.62–2.21)	1.81 (1.00–3.33)	0.12
Model II	1.00	1.21 (0.64–2.30)	1.83 (1.00–3.37)	0.12
Model III	1.00	1.33 (0.69–2.59)	2.13 (1.15–4.06)	0.052
Model IVa	1.00	1.31 (0.68–2.55)	2.16 (1.16–4.12)	0.05
Model IVb	1.00	1.23 (0.63–2.41)	1.83 (0.97–3.55)	0.16
Polyunsaturated fatty acid				
Median (g/d)	5.3	9.1	14.2	
Model I	1.00	0.95 (0.52–1.75)	1.10 (0.61–1.99)	0.90
Model II	1.00	0.98 (0.53–1.80)	1.08 (0.60–1.97)	0.94
Model III	1.00	0.97 (0.52–1.82)	1.12 (0.61–2.10)	0.88
Model IVa	1.00	0.99 (0.53–1.87)	1.18 (0.63–2.22)	0.83
Model IVb	1.00	0.91 (0.48–1.71)	1.03 (0.55–1.94)	0.92

^1^Values are medians and intakes were calculated as the percentage of energy by tertiles. OR and 95% CI calculated by logistic regression. Model I was crude model. Model II was additionally adjusted for age, pregravid BMI, and parity. Model III was additionally adjusted for family history of diabetes (yes or no), current smoking (yes or no), family income, total energy intake, gestational weight gain, and physical activity. Model IV a was additionally adjusted for carbohydrate intake. Model IV b was additionally adjusted for animal protein intake

### Sensitivity analysis

When we used total, specific, and source of fats estimated by FFQ to examine their association between total, specific, and source of fats and triglycerides (TG) and total cholesterol (TC), relation estimates remained similar, showing that dietary fat one year before pregnancy, and during pregnancy derived in our study were relatively accurate (data not shown).

## Discussion

In the present analysis, consumption of a diet with higher total fat intake during 12–22 weeks of gestation, but not in early pregnancy or one year before pregnancy, was associated with risks for GDM in a Chinese population. However, this association was greatly attenuated when animal protein intake was adjusted for.

To interpret associations between dietary fat intake and GDM risk, each observation time window should be considered, as nutrient intake during pregnancy and nutritional status before conception both affect pregnancy outcomes [28]. Compared with dietary fat intake before conception and in the early pregnancy, a clear increase was observed in mid-pregnancy. This is in line with a previous prospective study among middle-aged Spain women [29]. The reason for this result may be due to the transform of personal values and beliefs in nutrition, dietary recommendations from health professionals, and physiological changes during pregnancy [30].

Not only the different kinds of carbohydrates affect blood glucose levels or insulin secretion differently, but also the quantity ingested and body fat influence the development of insulin resistance. The consumption of certain kinds of carbohydrates do produce different effects on blood glucose concentrations in metabolically healthy people, people with impaired glucose tolerance or patients with type 2 diabetes mellitus (T2DM) [31, 32]. Fat has little, if any, effect on blood glucose levels, although a high fat intake does appear to contribute to insulin resistance [33]. In addition, protein content in the diet may also affect insulin secretion, sensitivity, or resistance and thereby postprandial blood glucose concentration [34]. Protein content of the diet tends to remain fairly consistent, while carbohydrate and fat content varies [33]. Thus, when carbohydrate intake increases, fat intake usually decreases, and vice versa. To date, researches that analyzed the association between dietary fat intake one year before pregnancy and during pregnancy and GDM risk while taking into account the effect of carbohydrate and protein are scarce. Although the precise pathological mechanisms underlying the association between macronutrient and GDM risks are still unclear, the current findings indicated that the protein intake, rather than dietary fat, might be the important macronutrient on the development of GDM, particularly those with normal BMI, low, or normal calorie intake.

We also observed that dietary total fat intake, neither one year before pregnancy nor during the first trimester of pregnancy, was correlated with the risk of developing GDM, which is in line with previous findings among 84,204 Nurses’ Health Study (NHS) women [35] or from 13,475 women in NHS II cohort study [22]. However, another study focusing on the quality of diet and the risk of GDM revealed that a tendency for increased fat intake was observed in the GDM groups among Caucasian women [21]. These inconsistent findings may be attributed to the racial differences in the regulation of fat and glucose metabolism [36]. In addition, food sources of fat may clarify the inconsistent role of dietary fat intake for incidence of GDM. Mayonnaise or salad dressing, whole milk, French fries, and fried potatoes, biscuits or muffins, and cheeses are the top five source fat in black and white pregnant women [37]. In contrast, cooking oil, red meat, and dairy products are major contributors to fat intake in Chinese women [16]. Interestingly, the red meat and dairy product are also major food sources of dietary animal protein, indicating that the impact of dietary fat on GDM may be mediated by animal protein, which was found in our analysis.

Fatty acids may play a vital role in glucose homeostasis [38]. However, the findings on the relation of these fatty acids with GDM are less consistent. Similar to our results, previous findings on a relevance of saturated fatty acid for GDM showed no significant association for both 1733 women [18] and 13,475 women [22] from U.S. The other studies in China and Canada consistently demonstrated that monounsaturated fatty acid [16] and polyunsaturated fatty acid [20] was not associated with GDM, while a prospective effect of SFA [19], MUFA [21] and PUFA [19] on GDM risk was observed in two reports from Europe. Some of the inconsistencies may be due to different study population characteristics, such as age, and underlying insulin resistance resulting from inactivity and excess gestational weight gain [39]. Methodological limitations could have also contributed to discrepant findings, such as absence of adjustment for dietary and nondietary factors. The availability of abundant information on covariates allowed to diminish potential confounders as much as possible [18, 22]. In addition, the inconsistencies could also be due to the differences in the dietary assessment methods. More recent studies [17, 20, 22], using validated FFQs and with large sample size, are more powerful to examine the association between diet fat and GDM.

The precise pathological mechanisms underlying the association between high dietary cholesterol consumption and glucose homeostasis and diabetes risks are still unclear. Our results showed that no significant association between women's total dietary cholesterol intake and the risk of GDM, which is in line with a case–control study conducted among 462 pregnant Iranian women [40]. However, the association between dietary cholesterol and GDM was not consistent in the NHS II cohort study [22]. In the current study, the average of dietary cholesterol intake among pregnant women was 362.0, 459.2 and 497.9 mg/d one year before conception and during the 1st and 2nd trimester, respectively, which was higher than that reported in pregnant women from NHS II cohort study. In addition, although Asian and Western population have similar sources of dietary cholesterol, the proportion of dietary cholesterol sources is very different: egg is the main sources of dietary cholesterol among Chinese adults while it is mainly derived from meats among US adults [41]. It may also lead to the inconsistence of our findings with studies in western countries [22].

The strengths of our study include the large sample size, the prospective study design, a high response rate, the uniform criteria for diagnosis of GDM, and more importantly, the availability of abundant information on dietary and nondietary covariates that allowed us to diminish potential confounders as much as possible. Notably, the detailed prospective assessment of the pregnancy diet with a validated FFQ might better reflect the habitual dietary intake over a period and a diagnosis of GDM could not affect dietary reporting. Finally, a key strength is the focus on the time period ranging from the year preceding pregnancy to birth, which makes it possible to investigate the critical time window of impact of dietary fat intake on GDM risk.

Several limitations in the current study should be mentioned. As in other observational studies, the possibility of unmeasured or unknown covariates might have led to residual confounding. In addition, nutrient intakes measured by FFQs are subject to measurement error. Nevertheless, because of the prospective design, trained investigators in our cohort, the use of standard serving bowls, plates, and glasses, food models representing standard portion size and a color photo book containing foods commonly consumed by Chinese adults, the estimation was more accurate and measurement error was reduced.

In conclusion, our analysis indicated that dietary fat intake in the year preceding pregnancy and during the first and second trimesters of pregnancy has no relevance on GDM risk, particularly in patients with normal BMI, low, or normal calorie intake, and macronutrients composition. The protein intake, rather than dietary fat, might be the important macronutrient on the development of GDM.
